# 1219. Unfavorable Clinical Outcomes with Polymyxins Compared to Ceftolozane/Tazobactam for the Treatment of Carbapenem-Resistant *Pseudomonas aeruginosa*

**DOI:** 10.1093/ofid/ofab466.1411

**Published:** 2021-12-04

**Authors:** Jessica Howard-Anderson, Michelle Earley, Toshimitsu Hamasaki, Chris W Bower, Gillian Smith, David van Duin, Scott R Evans, Jesse T Jacob

**Affiliations:** 1 Emory University, Decatur, GA; 2 George Washington University, Rockville, Maryland; 3 The George Washington University, Rockville, Maryland; 4 Georgia Emerging Infections Program, Decatur, GA; 5 University of North Carolina, Chapel Hill, North Carolina

## Abstract

**Background:**

Patients with carbapenem-resistant *Pseudomonas aeruginosa* (CRPA) have high in-hospital mortality rates. It is unknown if patients with CRPA treated with ceftolozane/tazobactam (C/T) have improved clinical outcomes compared to those treated with polymyxins.

**Methods:**

The CDC-funded, Georgia Emerging Infections Program performed active population- and laboratory-based surveillance for CRPA isolated from sterile sites, urine, lower respiratory tract and wounds in metropolitan Atlanta, GA from 8/1/2016–7/31/2018. We reviewed charts of adults without cystic fibrosis who were hospitalized within 1 week of CRPA culture. Using a desirability of outcome ranking (DOOR) analysis which incorporates both benefits and risks into a single outcome, we estimated the probability that a patient treated first with C/T would have a more desirable clinical outcome at 30-days than a patient treated with polymyxins (polymyxin B or colistin). We adjusted for confounding using inverse probability of treatment weighting (IPTW) based on culture source and need for dialysis at baseline. A partial credit analysis allowed for variable weighting of DOOR ranks and calculation of differences in mean partial credit scores.

**Results:**

Among 710 cases from 18 different hospitals, we identified 73 patients treated for CRPA infections with polymyxins (n=31) or C/T (n=42). Most patients were male (64%) and Black (80%), and those receiving polymyxins were more likely to have required dialysis at baseline (35% vs. 14%, p=0.03) (Table 1). At 30 days after culture, 34 (47%) were alive with no adverse events, 21 (29%) were alive with ≥ 1 adverse event, and 18 (25%) had died. Patients first treated with C/T had a lower 30-day mortality rate than those treated with polymyxins (14% vs 39%, p=0.03). Additionally, those receiving C/T had better overall clinical outcomes, with an adjusted DOOR probability of having an improved outcome of 67% (95% CI 53%–80%) compared to those receiving polymyxins (Figure 1). Partial credit analyses indicated consistent results across different patient values of survival with adverse events (Figure 2).

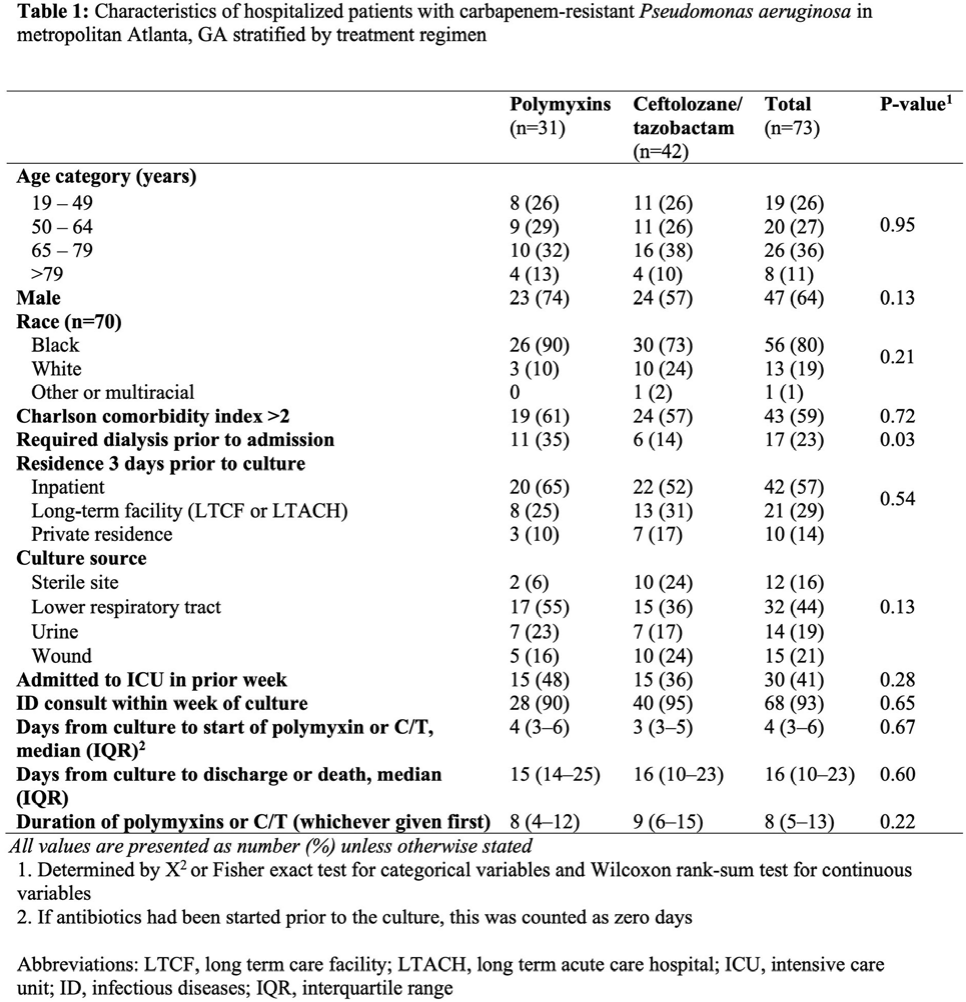

Figure 1: Inverse probability of treatment weighting-adjusted desirability of outcome ranking (DOOR) distributions by treatment group, accounting for adverse events and survival status that occurred up to 30 days after CRPA culture.

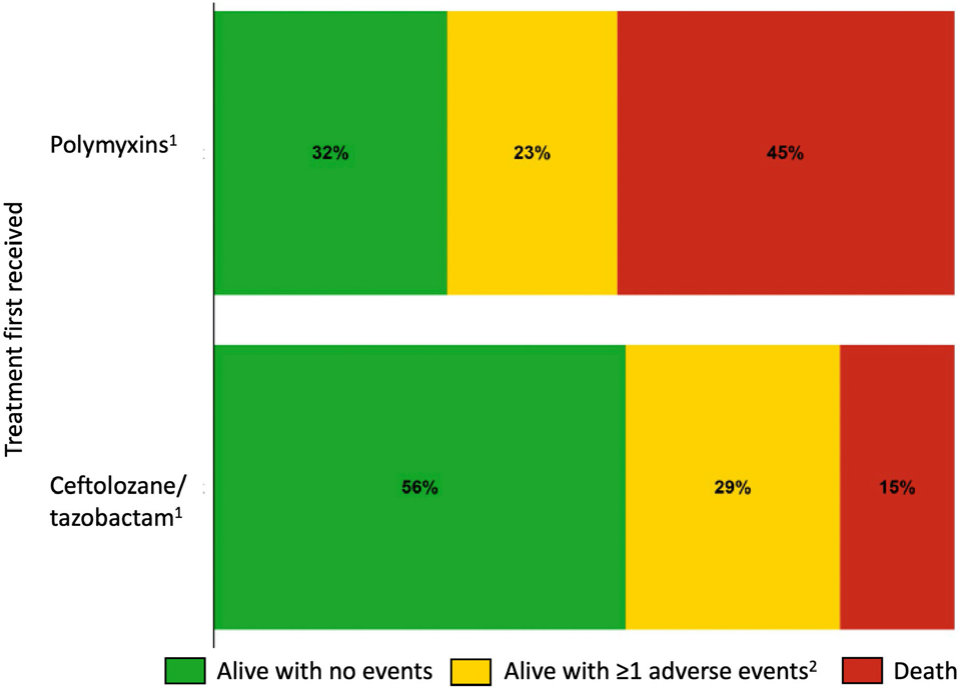

1. Percentages are adjusted using inverse probability of treatment weighting, controlling for culture source and need for dialysis at baseline 2. Adverse events measured included: acute kidney injury, discharge to higher acuity location than previous residence, or being hospitalized 30 days after culture

Figure 2: Inverse probability of treatment weighting-adjusted partial credit analysis.

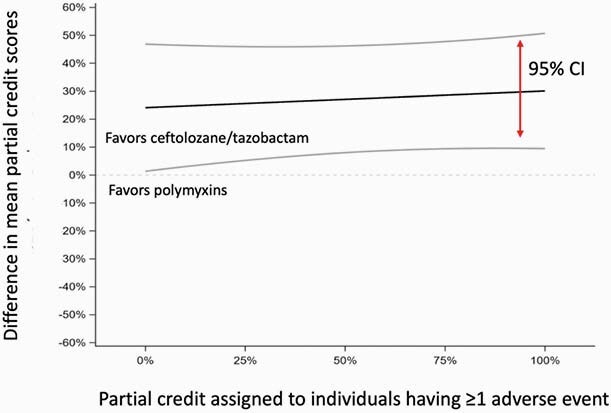

This displays the difference (ceftolozane/tazobactam minus polymyxin) in mean partial credit scores (black line) and associated 95% confidence bands (gray lines) as a function of the partial credit score assigned to an individual having at least one adverse event (range 0 – 100%). A score of 100% is assigned to patients alive with no adverse events and a score of 0% is assigned to patients who die. A difference in mean partial credit scores of approximately zero suggests there was no difference observed between treatment groups.

**Conclusion:**

These findings support the recent Infectious Diseases Society of America guidance favoring C/T over polymyxins for treatment of CRPA infections.

**Disclosures:**

**David van Duin, MD, PhD**, **Entasis** (Advisor or Review Panel member)**genentech** (Advisor or Review Panel member)**Karius** (Advisor or Review Panel member)**Merck** (Grant/Research Support, Advisor or Review Panel member)**Pfizer** (Consultant, Advisor or Review Panel member)**Qpex** (Advisor or Review Panel member)**Shionogi** (Grant/Research Support, Scientific Research Study Investigator, Advisor or Review Panel member)**Utility** (Advisor or Review Panel member) **Scott R. Evans, PhD**, **Abbvie** (Consultant)**Advantagene** (Consultant)**Alexion** (Consultant)**Amgen** (Consultant)**AstraZeneca** (Consultant)**Atricure** (Consultant)**Breast International Group** (Consultant)**Cardinal Health** (Consultant)**Clover** (Consultant)**FHI Clinical** (Consultant)**Genentech** (Consultant)**Gilead** (Consultant)**Horizon** (Consultant)**International Drug Development Institute** (Consultant)**Lung Biotech** (Consultant)**Microbiotix** (Consultant)**Neovasc** (Consultant)**Nobel Pharma** (Consultant)**Novartis** (Consultant)**Nuvelution** (Consultant)**Pfizer** (Consultant)**Rakuten** (Consultant)**Roche** (Consultant)**Roivant** (Consultant)**SAB Biopharm** (Consultant)**Shire** (Consultant)**Stryker** (Consultant)**SVB Leerink** (Consultant)**Takeda** (Consultant)**Teva** (Consultant)**Tracon** (Consultant)**Vir** (Consultant)

